# Supplementary material for: Predictive performance of clinical scores and survival outcomes in critically ill patients with sepsis: a prospective longitudinal study at a tertiary medical centre in Ethiopia
Source: PeerJ. 2025 Oct 2;13:e20109. doi: 10.7717/peerj.20109 (PMC12497402; doi:10.7717/peerj.20109)
Supplement: Supplemental Information 1 [file peerj-13-20109-s001.docx]

**Table** **1**. Variables and score points to calculate clinical scores

| **SIRS** | | | | | | | |
| --- | --- | --- | --- | --- | --- | --- | --- |
| **Variables** | **0 point** | | | | **1 point** | | |
| Temperature (^0^c) | 36.0-38.0 | | | | >38.0or <36.0 | | |
| RR (breaths/min) | <20 | | | | >20 | | |
| HR (beats/min) | <90 | | | | >90 | | |
| WBC count (cells/mm^3^) | 4,000-12,000 | | | | >12,000 or <4,0000 | | |
| **qSOFA** | | | | | | | |
| **Variables** | **0 point** | | | | **1 point** | | |
| GCS | 15 | | | | <15 | | |
| RR (breaths/min) | <22 | | | | >22 | | |
| SBP mmHg) | >100 | | | | <100 | | |
| **UVA** | | | | | | | |
| **Variables** | **0 point** | | **1point** | **2 points** | **3 points** | **4 points** | |
| Temperature (^0^c) | >36.0 | |  | <36.0 |  |  | |
| HR (beats/min) | <120 | | >120 |  |  |  | |
| RR (breaths/min) | **<30** | | **>30** |  |  |  | |
| SBP mmHg) | **>90** | | **<90** |  |  |  | |
| Oxygen saturation (%) | **>92** | |  | **<92** |  |  | |
| GCS | **15** | |  |  |  | **<15** | |
| HIV infection | **No/Unknown** | |  | **Yes** |  |  | |
| **MEWS score** | | | | | | | |
| **Variables** | **3 pts.** | **2 pts.** | **1point** | **0 point** | **1 point** | **2 points** | **3 points** |
| RR (breaths/min) |  | <8 |  | 9-14 | 15-20 | 21-29 | >29 |
| HR (beats/min) |  | <40 | 41-50 | 51-100 | 101-110 | 111-129 | >129 |
| SBP mmHg) | <70 | 71-80 | 81-100 | 101-199 |  | >200 |  |
| Temperature (^0^c) |  | <35 | 35.1-36.0 | 36.1-38.0 | 38.1-38.5 | >38.6 |  |
| AVPU |  |  |  | A | V | P | U |
| **NEWS Score** | | | | | | | |
| **Variables** | **3 pt.** | **2 pt.** | **1pt.** | **0 pt.** | **1 pt.** | **2 pt.** | **3 pt.** |
| RR (breaths/min) | <8 |  | 9-11 | 12-20 |  | 21-24 | >25 |
| Oxygen saturation (%) | <91 | 92-93 | 94-95 | >96 |  |  |  |
| Any supplemental oxygen |  | Yes |  | No |  |  |  |
| Temperature (°C) | <35 |  | 35.1-36.0 | 36.1-38.0 | 38.1-39 | >39.1 |  |
| SBP (mmHg) | <90 | 91-100 | 101-110 | 111-219 |  |  | >220 |
| HR (beats/min) | <40 |  | 41-50 | 51-90 | 91-110 | 111-130 | >131 |
| AVPU scale |  |  |  | A |  |  | V, P, or U |
