# Supplementary material for: Predictive performance of clinical scores and survival outcomes in critically ill patients with sepsis: a prospective longitudinal study at a tertiary medical centre in Ethiopia
Source: PeerJ. 2025 Oct 2;13:e20109. doi: 10.7717/peerj.20109 (PMC12497402; doi:10.7717/peerj.20109)
Supplement: Supplemental Information 4 [file peerj-13-20109-s004.docx]

**Code book**

Education: 1=no formal education; 2=primary school; 3=secondary; 4=college and above

Employment: 1=employed; 2= unemployed

Marital status: 1= married; 2=single; 3= divorced; 4= widowed

Clinical suspicion of sepsis: 1=yes; 2= no

Diagnostic category: 1=medical; 2= surgical

Site of infection: 1= lung; 2= pleura; 3=abdomen; 4=urinary tract; 5=blood stream; 6= soft tissue; 7= CNS; 8= other

Comorbidities: 1= respiratory; 2=HIV; 3= TB; 4= malaria; 5= CVD; 6= HTN; 7= DM; 8= CRF; 9= cancer; 10= cirrhosis; 11= others

What is done for the patient: 1= mechanical ventilation; 2= vasopressor; 3= antibiotics; 4= food; 5= other

Organ failure: 1= liver; 2= kidney; 3= lungs; 4== heart; 5= brain; 6= other

Stay before ICU admission: 1= emergency room; 2= internal medicine; 3= surgery; 4= orthopedics; 5= OBY-GYN; 6= pediatrics ward; 7= other

Surgical status: 1= elective; 2= no surgery; 3= emergency
